# Effect of ambient temperature on emergency department visits in Shanghai, China: a time series study

**DOI:** 10.1186/1476-069X-13-100

**Published:** 2014-11-25

**Authors:** Yue Zhang, Chenyang Yan, Haidong Kan, Junshan Cao, Li Peng, Jianming Xu, Weibing Wang

**Affiliations:** School of Public Health & Key Laboratory of Public Health Safety (Ministry of Education), Fudan University, Shanghai, China; Fudan Tyndall Centre, Shanghai, China; Department of teaching and medical administration, Changzheng Hospital, Second Military Medical University, Shanghai, China; Shanghai Municipal Human Resources and Social Security Bureau, Shanghai, China; Shanghai Center for Urban Environmental Meteorology, Shanghai, China; Shanghai Key Laboratory of Meteorology and Health, Shanghai, China

**Keywords:** Ambient temperature, Emergency department visits, Generalized additive model

## Abstract

**Background:**

Many studies have examined the association between ambient temperature and mortality. However, less evidence is available on the temperature effects on gender- and age-specific emergency department visits, especially in developing countries. In this study, we examined the short-term effects of daily ambient temperature on emergency department visits (ED visits) in Shanghai.

**Methods:**

Daily ED visits and daily ambient temperatures between January 2006 and December 2011 were analyzed. After controlling for secular and seasonal trends, weather, air pollution and other confounding factors, a Poisson generalized additive model (GAM) was used to examine the associations between ambient temperature and gender- and age-specific ED visits. A moving average lag model was used to evaluate the lag effects of temperature on ED visits.

**Results:**

Low temperature was associated with an overall 2.76% (95% confidence interval (CI): 1.73 to 3.80) increase in ED visits per 1°C decrease in temperature at Lag1 day, 2.03% (95% CI: 1.04 to 3.03) and 2.45% (95% CI: 1.40 to 3.52) for males and females. High temperature resulted in an overall 1.78% (95% CI: 1.05 to 2.51) increase in ED visits per 1°C increase in temperature on the same day, 1.81% (95% CI: 1.08 to 2.54) among males and 1.75% (95% CI: 1.03 to 2.49) among females. The cold effect appeared to be more acute among younger people aged <45 years, whereas the effects were consistent on individuals aged ≥65 years. In contrast, the effects of high temperature were relatively consistent over all age groups.

**Conclusions:**

These findings suggest a significant association between ambient temperature and ED visits in Shanghai. Both cold and hot temperatures increased the relative risk of ED visits. This knowledge has the potential to advance prevention efforts targeting weather-sensitive conditions.

**Electronic supplementary material:**

The online version of this article (doi:10.1186/1476-069X-13-100) contains supplementary material, which is available to authorized users.

## Background

The association between short-term exposure to high or low ambient temperatures and increased mortality has been well documented worldwide [1, 2]. Studies have assessed associations with specific categories of health outcomes, such as respiratory diseases [3, 4], identifying more immediate, acute effects as well as vulnerabilities among specific populations. However, it is also useful to study emergency department (ED) visits broadly, as they may provide insight into the mechanisms involved in the effects of temperature on health and may assist in the development of appropriate interventions [5]. Even determining the predictors of vulnerability to heat effects would help improve health education and interventions targeting those who are most susceptible.

While most studies used mortality as the main health outcome, fatal events constitute only a portion of acute health effects. Thus non-fatal morbidities such as outpatient visits or hospitalizations should also be considered in adequately characterizing the health effects of exposure to low or high temperatures [6]. Less is known about the association between ambient temperature and morbidity, with most evidence coming from studies of hospital visits [7] and much less from studies of ED visits [3]. Currently, the sources of health indicator data related to extreme weather exposure are primarily hospital-based. Heat-related increases in respiratory admissions have been observed, particularly in elderly people [4]. Other studies have focused specifically on ED data. For example, the Paris Index of Heat stroke Related Excess Mortality was formulated to warn of mortality increases during heat waves [8]. A study from Italy showed a strong relationship between bio-meteorological conditions and ambulance dispatches [9].

Current knowledge about the effects of ambient temperature on ED visits derives mainly from developed western countries [3, 10]. Few studies have been conducted in developing countries such as China, despite their populations being more sensitive to changes in weather conditions due to their poorer public health infrastructure and more vulnerable populations [11]. Few Chinese cities have established city-wide morbidity reporting systems, limiting the availability of data on the association between ambient temperature and morbidity outcomes. Shanghai has a moderate subtropical climate, with four distinct seasons and a higher rate for emergency department visits. We therefore performed a time-series analysis evaluating the exposure-response relationship between ED visits and ambient temperature in Shanghai, the largest city in China. A better understanding of the possible interaction will provide relevant information for developing public health plans and risk assessments in an ambient environment.

## Methods

### Study population

Shanghai is the most populous city in China, with a total population of over 23 million people in 2010, of which 14 million are registered residents. We obtained daily number of ED visits for registered residents living in urban Shanghai who participated in workers’ basic medical insurance, including employees of urban businesses, organizations, institutions, and social organizations between January 1, 2006 and December 31, 2011 from the Shanghai Health Insurance Bureau (SHIB). Our analysis covers approximate seven million permanent residents living in all nine urban districts (279 square kilometers) of Shanghai (Additional file 1: Figure S1). The SHIB is a government agency that administers the Shanghai Health Insurance System. The Shanghai Health Insurance System, which provides compulsory universal health insurance and the proportion of registered residents who participated in urban workers’ basic medical insurance was 70.4%. All hospitals are under contract with this system and computerized records of hospital visits are maintained at each contracted hospital and sent to the SHIB through an internal computer network.

We defined a case as any ED visit by an individual on a given day from one midnight to the next. Patients were not assigned unique identifiers, so any ED visit during the study period was considered a separate case. Any ED visit, regardless of whether the patient was treated and released, admitted to the hospital, or died in the ED, was included in the analysis.

### Meteorological and air pollutant data

Meteorological data on daily minimum, maximum and mean temperatures (°C), relative humidity (%), rainfall (mm), wind speed (m/s) were obtained from the Shanghai Center for Urban Environmental Meteorology. The weather data were measured at a fix-site station located in Xuhui District of Shanghai. Air pollution data included PM_10–2.5_ (the fraction of particulates in air of very small size (2.5 μm < PM_10–2.5_ < 10 μm)), sulfur dioxide (SO_2_) and nitrogen dioxide (NO_2_) during the same period were obtained from Shanghai Environmental Monitoring Center. The city-wide daily mean concentrations for each pollutant were averaged from the available data of six fixed-site monitoring stations (Hongkou, Jin’an, Luwan, Putou, Xuhui and Yangpu).

Patient records/information was de-identified prior to analysis; then daily aggregated counts for ED visits were calculated and used to conduct the final analysis. The authors did not access to patient individual information prior to anonymization and data aggregation, and there was no any interaction with the patients for this study. An exempt from al review have been approved by the Ethics Committee of School of Public Health, Fudan University.

### Data analysis

As the number of daily hospital admissions is a type of low probability event and typically follows a Poisson distribution [12], the semi-parametric generalized additive model (GAM) approach with log link was used to explore the association between ambient temperature (DMT) and daily ED visits, accounting for any overdispersion or autocorrelation. In some studies, daily maximum or minimum temperature, synthetic measure such as humidex or apparent temperature were used to examined the effects on EDVs. Daily measurements may have different impacts with average ambient temperature on EDVs. Sensitivity analyses from previous studies using multiple temperature metrics have found that the effect estimates of all temperature metrics were similar [13, 14]. Moreover, considering daily mean temperature may be able to provide more easily interpreted results in a policy context and more familiar to the public, we use the ambient temperature for subsequent analyses.

First, we utilized a time-series model to assess the relationship between ED visits and ambient temperature, while controlling for air pollutants (PM_10–2.5_, SO_2_ and NO_2_), relative humidity and wind speed. We controlled long-term trends and seasonal patterns, as well as meteorological factors using smoothed spline functions, which can accommodate nonlinear and non-monotonic patterns between the number of ED visits and time or weather conditions [15]. Day of the week (DOW) and public holidays (Holiday) were adjusted as dummy variables in the model. Residuals of the basic models were used to check whether there were discernible patterns and autocorrelation by means of residual plots and partial autocorrelation function plots [16]. Specifically, the degrees of freedom (df) per year for time trends were defined for ED visits (df = 4). For weather conditions, the selection of degrees of freedom was based on minimizing Akaike’s Information Criterion (AIC). Model selection for the other confounders was carried out by minimizing the Generalized Cross Validation (GCV) criterion. The basic model was calculated as:

Where Y_t_ refers to the number of observations; .E(Y_t_) denotes the estimated ED visits on day t; α is the intercept; γ is the vector of coefficients for DMT_t_; S() denotes a regression spline function for nonlinear variables; time is the number of calendar days on day t; df is the degrees of freedom; DOW is the day of the week; Holiday denotes public holidays; and Z_t_ indicates pollutant concentrations on day t.

Second, we plotted the exposure-response curve between ED visits and ambient temperature based on the GAM. If this relationship was linear or almost linear, temperature effects were directly presented as relative risk (RR) of ED visits per 1°C change in temperature. If the relationship was non-linear, we selected a temperature breakpoint (commonly called the “threshold”) and used a linear-threshold model to quantify the effect of ambient temperature [17]. Temperature effects were reported as the RR of ED visits per 1°C change in temperature above (hot effects) or below (cold effects) the threshold, with excess risk (ER) calculated as RR minus1.

Since temperature can not only affect hospital admissions on the current day but on several subsequent days (lag effect) [18, 19], we used a moving average lag model to evaluate the lag effects. For example, a lag of 0 days represents the current-day temperature; a lag of 1 indicates a 2-day moving average of current and previous day temperatures.

After assessing the effects of ambient temperature on ED visits for the entire population, we repeated the same procedure to examine associations stratified by gender and age (<45, 45–65, 65–75 and ≥75 years).

All statistical analyses were performed using the R statistical environment (version 3.0.1) with the “mgcv” package used to fit the regression model.

## Results

### Data description

ED visits distributions by gender and age are summarized in Table 1. There were totally 23,014,261 ED visits during 2006 to 2011, including 10,254,175 (44.6%) male and 12,760,086 (55.4%) female. There was an average of 10,504, 4680 and 5824 for all, male and female, respectively. The average number of ED visits by age was 2961 (28.2%), 4147 (39.5%), 1308 (12.5%) and 2088 (19.8%) for age groups (<45,45-65, 65–75, and ≥75 years old). Table 2 shows the basic statistics for weather conditions and air pollution. During the same time period, the mean daily concentrations of PM_10–2.5_, SO_2_ and NO_2_ were 81.95, 41.05 and 55.46 μg/m^3^, respectively. The mean daily average temperature was 17.54°C, and mean relative humidity was 69.38%, reflecting the subtropical climate in Shanghai.

**Table 1:** **Summary statistics of daily emergency visits in Shanghai, 2006-2011**

	Total EDVs		Daily EDVs	
	Number	%	Mean	SD^a^
All	23014261		10504	2398
Gender				
Male	10254175	44.6	4680	1071
Female	12760086	55.4	5824	1336
Age				
<45	6487841	28.2	2961	845
45-65	9085851	39.5	4147	955
65-75	2866628	12.5	1308	240
≥75	4573932	19.8	2088	565

EDVs: emergency department visits; ^a^Standard deviation.

**Table 2:** **Summary of daily weather factors and air pollution concentrations in Shanghai, 2006-2011**

Daily data	Mean	SD	Min	P(25)	Median	P(75)	Max
Air pollution							
PM_10–2.5_ (ug/m^3^)	81.95	53.59	12.18	45.67	69.41	103.00	599.29
SO_2_ (ug/m^3^)	41.05	23.48	10.59	24	34.72	49.33	195.50
NO_2_ (ug/m^3^)	55.46	20.98	12.42	40.80	51.64	67.02	141.64
Weather condition							
DMT (°C)	17.54	9.03	−3.40	9.70	18.60	25.10	35.70
DLT (°C)	14.45	9.20	−6.80	6.60	15.00	22.60	31.80
DHT (°C)	21.20	9.33	0.50	13.50	22.30	28.80	39.40
RH (%)	69.38	11.90	23	62	70	79	95
JS (mm)	31.74	98.62	0	0	0	11.00	1284
WS (m/s)	2.97	1.02	0.40	2.20	2.80	3.60	8.70

DMT: daily mean temperature; DLT: daily lowest temperature; DHT: daily highest temperature; RH: relative humidity; JS: rainfall; WS: wind speed.

### Daily ED visits and ambient temperature

**Figure 1:**
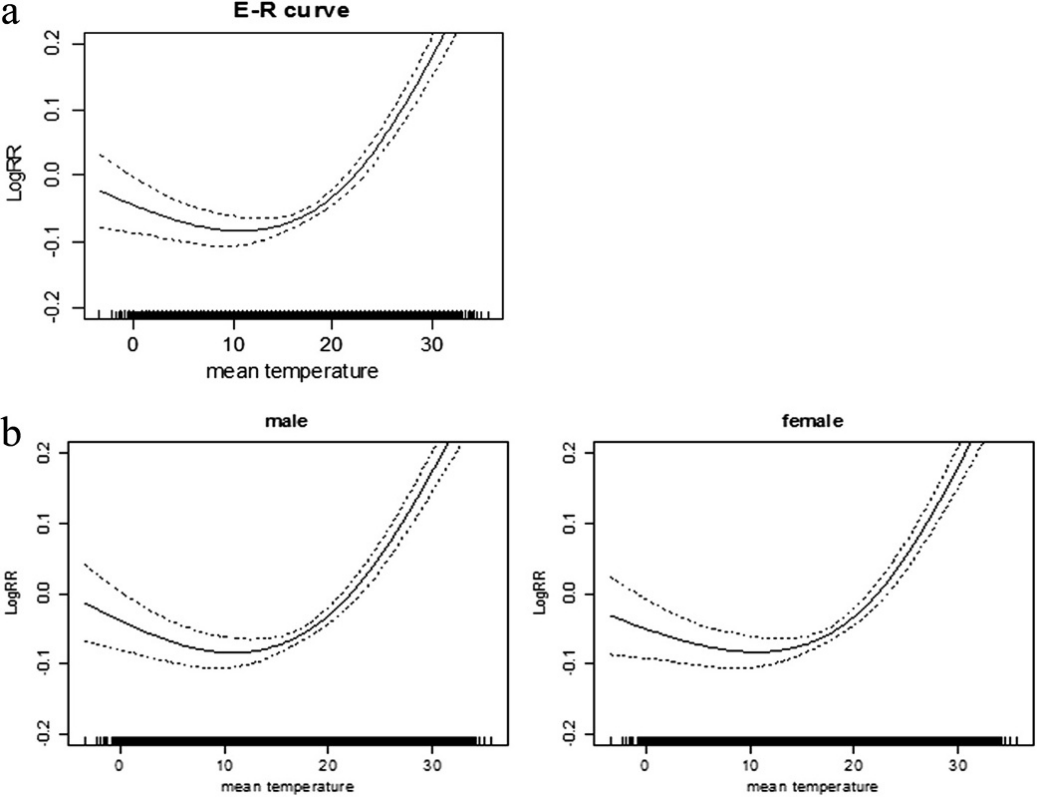
**Exposure-response curves of daily mean temperature (lag = 0) and daily emergency department visits. a)** among the entire study cohort; **b)** among males and females separately. The solid lines indicate the estimated mean percentage of change in ED visits, and the dotted lines represent twice the standard error.

Daily ED visits and temperature over time are presented in Figure 1, after adjusting for long-term and seasonal trends, weather, DOW, air pollution and other confounders. The relationship between mean temperature and ED visits followed a U-shaped curve, with the two being significantly associated (*P < 0.001*). The optimum temperature (OT), corresponding to the lowest point in the E-R curve, was about 12°C; the risk of ED visits increased at temperatures below the OT and then reversed the trend.

### Gender differences on the effects of ambient temperature

**Figure 2:**
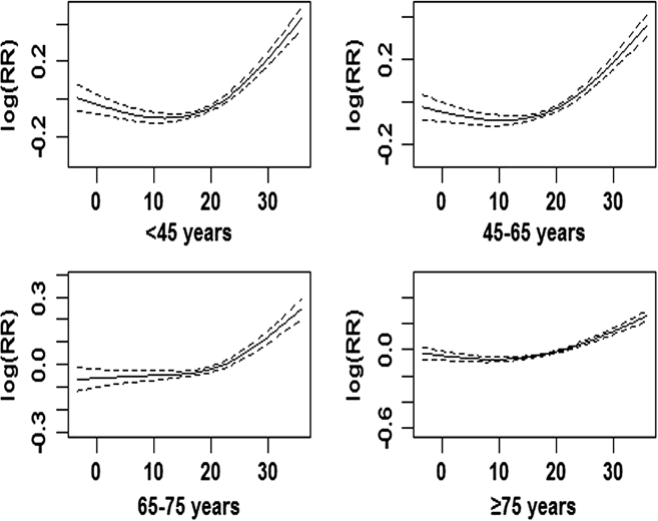
**Exposure-response relationship curves of ambient temperature versus emergency department visits by subject age.** The solid lines show mean estimates and the dotted lines show 95% confidence intervals.

The exposure-response analysis showed similar risk estimates with mean temperatures in males and females, with similar ED visit-ambient temperature curves and OTs of 12°C (Figure 2). Table 3 shows that the magnitude of the effects of daily mean temperature varied by gender. The effect of temperature was significant with a 0–3 day lag structure in the multi-day lag models. Significant associations were found between temperature and ED visits among both males and females. The cumulative effects of cold temperature reached a maximum after a lag of one day in the overall population and separately among males and females. In contrast, the cumulative effects of high temperature reached a maximum on the current day in all three cohorts. For cold effects, a 1°C decrease in ambient temperature after a lag of one day was associated with an overall 2.76% (95% CI: 1.73 to 3.80) increase in ED visits, 2.03% (95% CI: 1.04 to 3.03)among males and 2.45% (95% CI: 1.40 to 3.52) among females. In contrast, a 1°C increase in ambient temperature on the same day resulted in an overall 1.78% (95% CI: 1.05 to 2.51) increase in ED visits, 1.81% (95% CI: 1.08 to 2.54) among males and 1.75% (95% CI: 1.03 to 2.49) among females.

**Table 3:** **Percent increase in and relative risk of daily ED visits associated with a 1°C change in the moving average ambient temperature, with adjustment for the time trend, DOW, holiday, humidity, rainfall, wind speed and air pollution (SO**_**2**_ **, NO**_**2**_ **,PM**_**10–2.5**_ **)**

			Effects of cold			Effects of heat		
	OT	Lag	ER (%)	RR	95% CI of ER	ER (%)	RR	95% CI of ER
All	12	0	2.06*	1.0206	(1.07-3.07)	1.78*	1.0178	(1.05-2.51)
		1	2.76*	1.0276	(1.73-3.80)	1.42*	1.0142	(0.62-2.22)
		2	1.33*	1.0133	(0.28-2.40)	1.29*	1.0129	(0.43-2.17)
		3	1.09*	1.0109	(0.04-2.15)	1.18*	1.0118	(0.28-2.09)
Males	12	0	1.88*	1.0188	(0.91-2.86)	1.81*	1.0181	(1.08-2.54)
		1	2.03*	1.0203	(1.04-3.03)	1.46*	1.0146	(0.65-2.27)
		2	1.07*	1.0107	(0.05-2.11)	1.31*	1.0131	(0.44-2.10)
		3	0.83	1.0083	(−0.19-1.86)	1.18*	1.0119	(0.27-2.10)
Females	12	0	2.21*	1.0221	(1.18-3.25)	1.75*	1.0175	(1.03-2.49)
		1	2.45*	1.0245	(1.40-3.52)	1.39*	1.0139	(0.59-2.19)
		2	1.53*	1.0153	(0.45-2.62)	1.28*	1.0128	(0.42-2.15)
		3	1.30*	1.0130	(0.21-2.40)	1.18*	1.0118	(0.41-2.15)

*P-value < 0.05; OT: optimum temperature as the cut-off point for low and high temperature; ER: excess risk; RR: relative risk.

### Age stratified analysis of the effects of ambient temperature

**Figure 3:**
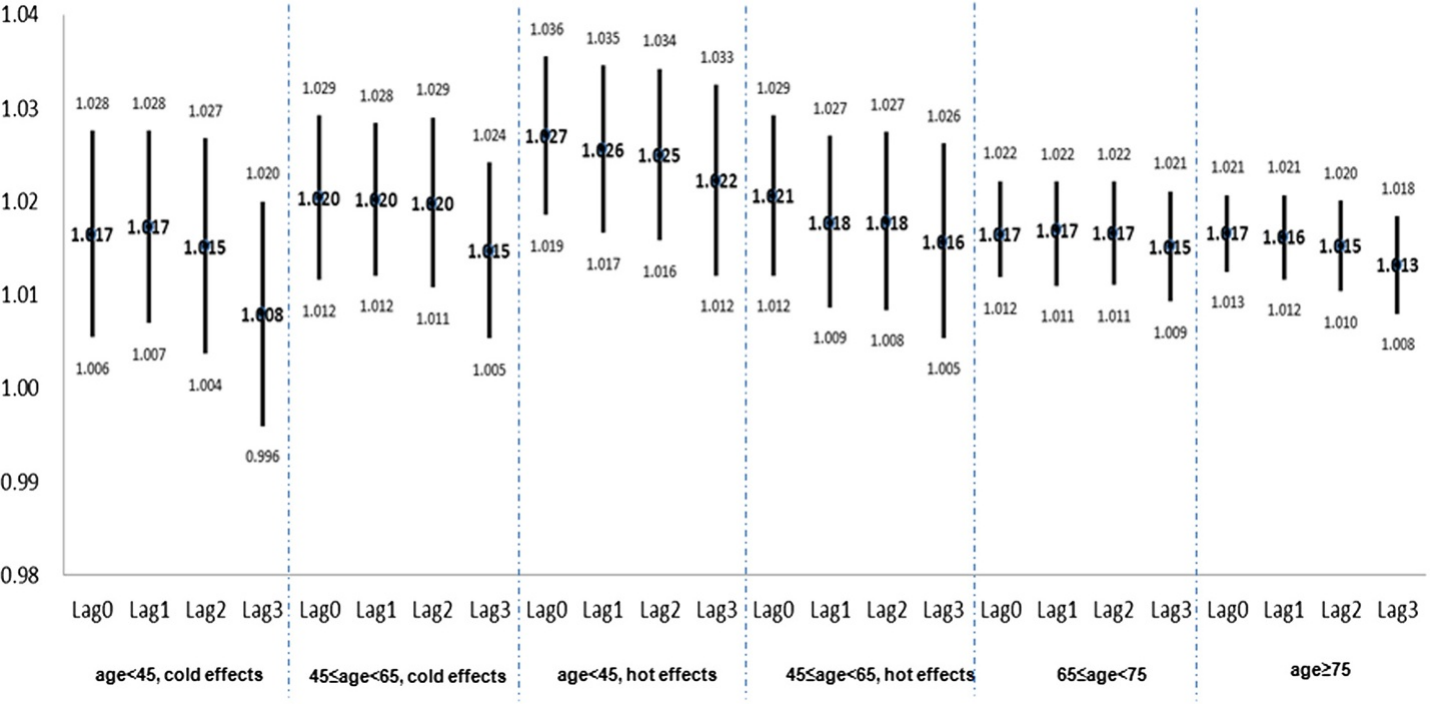
**Relative risks of ED visits associated with a 1°C change in temperature by age groups in Shanghai.** OT: optimum temperature as the cut-off point for low and high temperature; ED: emergency department visits.

All age groups showed a statistically significant (*P* < 0.05) association between the log(RR) of ED visits and ambient temperature (Figure 3).The population aged <65 years showed an OT of 15°C, but no OT could be determined for those aged ≥65 years as the curve showed a liner trend. The effects of cold (ER at a lag of one day) were greater for people aged 45–64 years than for those aged <45 years (2.04% vs. 1.73%), whereas the effects of heat (ER on the same day) were greater for persons aged <45 years (2.71%, 95% CI: 1.86 to 3.55) than those aged 45–64 years (2.06%, 95% CI: 1.20 to 2.90). For people aged >65 years, a 1°C increase in ambient temperature was associated with a 1.65% (95% CI: 1.20 to 2.20) increase in ED visits on the same day.

The lag effect was apparently age dependent. The cold effect appeared to be relatively acute in younger people, aged <45 years, but showed consistent effects in individuals aged ≥65 years. In contrast, the effects of higher temperature were relatively consistent over all age groups.

## Discussion

This time-series analysis showed that ambient temperature was associated with ED visits in Shanghai, followed a U-shape curve. The effect increased significantly when temperature fell below the threshold temperature. It is known that general V-, U- or J-shape relationship exist between daily mortality counts and temperature, with deaths increasing as temperatures fall below or rise above certain threshold values [20, 21]. These heat and cold slopes are usually quantified once the thresholds are estimated and fixed at specific values [22, 23]. A multicity study in Australia found that the temperature-mortality relations appeared to be a U shape across three cities [24]. Similar exposure-response relationships were found between temperature and emergency department visits, basically in ‘J’-shaped version. But the estimated spline curve of ED visits showed a higher threshold temperature [25].

We also observed a lag time of 0–3 days for assessing the cumulative effects of cold temperature on ED visits. The effects of cold ambient temperature on ED visits appeared to be relatively acute. Several previous studies found that both hot and cold temperatures had significant impacts on mortality rates in Shanghai [26, 27]. In the USA, Basu et al. found that increased temperatures had same-day effects on ED visits and age seemed to modify some of these impacts [10]. In Australia, Toloo et al. found emergency departments significantly increased on heatwave days and the RRs in different age groups ranged between 3.0 ~ 9.2 when two or more successive days with daily maximum temperature was ≥34°C [28]. In Shanghai, Sun et al. found heat wave with intensity above the 90^th^ percentile had 2.62% and 0.95% increases in ED visits for a duration of at least 2 days and 3 days respectively [25]. Although the relationship between ambient temperature and ED visits had previously been evaluated [29], to our knowledge, this is one of the few studies in China to evaluate the acute effects of ambient temperature over a 6-year period.

Short-term effects of ambient temperature and air pollution on mortality have been extensively evaluated in North American and European countries [30–32]. Studies in China identified a clear seasonal pattern in all-cause mortality, with a sharper spike in winter [33], and an association between short-term exposure to outdoor air pollution and an increased risk of ED visits in Shanghai in 2009 [26]. PM_2.5_ and PM_10–2.5_ were recently reported to show significant effects on daily ED visits in Shanghai [34]. Because of limitations in monitoring meteorological and morbidity data, however, few epidemiological studies in China have addressed associations between ambient temperature and ED visits, especially after adjustment for other meteorological factors and air pollutants. Moreover, to our knowledge, no previous study has evaluated these associations by gender and age.

Emergency departments are increasingly serving as the safety net, as the burden placed on them by the underserved population has been increasing, both in terms of the overall volume and types of illness that could potentially have been treated in primary care facilities [35]. We observed significant acute effects (within 0–3 days) of ambient temperature on daily visits to the ED. The underlying causes of this discrepancy were unclear. In contrast to outpatient visits, ED visits are less subject to these influencing factors, and are therefore considered a good indicator of the occurrence of illnesses [36].

We observed age-associated differences in exposure-response curves, with subjects aged ≥65 years being more sensitive to hot temperatures, whereas younger individuals were sensitive to both hot and cold. This is consistent with several morbidity studies in western countries showing that older persons were more vulnerable to high temperatures [37, 38]. This may be related to a reduced thermoregulatory capacity and decreased ability to detect changes in body temperatures of older persons, as well as their cognitive impairment and diminished mobility, which limit their behavioral defenses. In our study, the effects of heat on ED visits were greater for persons aged <45 years than those aged 45–64 years. Similar findings were observed in Beijing City, China [39]. This may be because persons aged <45 years belonged to occupational population who were more susceptible to variability of ambient temperature. In developed countries such as the USA, 15% of all ED visits were by persons aged ≥65 years [40]. As the proportion of older individuals in Shanghai continues to increase, so will their utilization of ED services.

The effect of ambient temperature on ED visits identified different lag structures, findings consistent with other studies of morbidity in Asian countries [26, 41]. We observed statistically significant associations for some, but not all, lag structures of ambient temperature. Further research is needed to clarify the lag structure and magnitude of such effects.

This study had several limitations. First, we used weather conditions at one meteorological station in Shanghai as measurements of ambient temperature rather than measures of personal exposure. The use of ambient rather than personal exposure measures may result in exposure misclassification. Moreover, the temperature difference between indoors and outdoors due to air conditioning or heating may affect the association between temperature and ED visits. Second, the morbidity data were from urban residents who registered the Shanghai Health Insurance System and most vulnerable groups (e.g., floating population and unemployed residents) may not involve in this study, thus possibly introducing a selection bias. Thirdly, this study didn’t assess the factors that may have association with ED visits, such as ozone, availability or ease of transportation, patients’ willingness to travel to the hospital, modifications from other socioeconomic status. Ozone is a potential confound factor in the relationship between temperature, particularly warm temperature, and all-cause ED visits, and not controlling for ozone in the analysis is likely to have resulted in overestimates of the observed association at high temperatures. Finally, many of the cases seen in ED visits are not temperature-sensitive. However, due to the unavailability of the cause-specific emergency department visits, we failed to explore the exposure-response association based on major subsets of outcomes, such as cardiovascular or respiratory diseases.

## Conclusions

We observed significant associations between exposure to both cold and hot ambient temperatures and increased ED visits in different gender and age groups in Shanghai, China. The effects of cold were stronger and more acute than those of heat, with both having different effects by gender and age. These results demonstrate that ambient temperature is an important environmental hazard in Shanghai. These findings could help establish public health preparedness and interventions to minimize adverse health effects of ambient temperatures.

## Electronic supplementary material

Additional file 1: Figure S1:**a)** Map of Shanghai (the red areas are the study sites); **b)** Locations of the weather monitoring station (in black) providing meteorological data and six monitoring stations (in green) providing air pollution data. (TIFF 579 KB)

## Abbreviations

ED: Emergency department
RR: Relative risk
ER: Excess risk
OT: The optimum temperature
CI: Confidence interval
DMT: Daily mean temperature
DLT: Daily lowest temperature
DHT: Daily highest temperature
RH: Relative humidity
JS: Rainfall
WS: Wind speed
SO_2_: Sulfur dioxide
NO_2_: Nitrogen dioxide.
