# Supplementary figures and images for: Effect of ambient temperature on emergency department visits in Shanghai, China: a time series study
Source: Environ Health. 2014 Nov 25;13:100. doi: 10.1186/1476-069X-13-100 (PMC4258028; doi:10.1186/1476-069X-13-100)

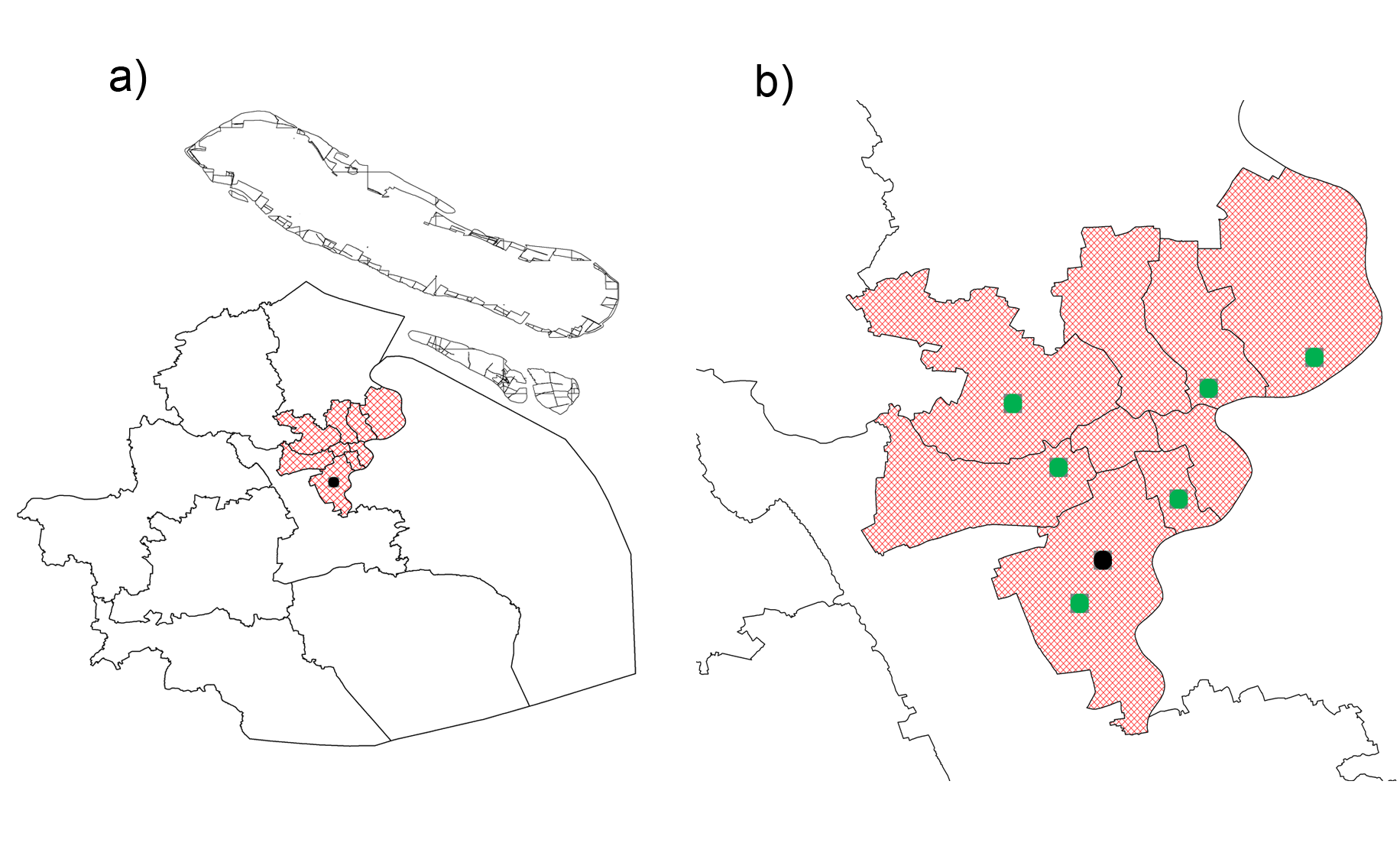

Supplement: Supplementary file 1 — Additional file 1: Figure S1:a) Map of Shanghai (the red areas are the study sites); b) Locations of the weather monitoring station (in black) providing meteorological data and six monitoring stations (in green) providing air pollution data. (TIFF 579 KB) [file 12940_2014_801_MOESM1_ESM.tiff]
